# Author Correction: The role of mountains in shaping the global meridional overturning circulation

**DOI:** 10.1038/s41467-024-49558-6

**Published:** 2024-06-21

**Authors:** Haijun Yang, Rui Jiang, Qin Wen, Yimin Liu, Guoxiong Wu, Jianping Huang

**Affiliations:** 1 Department of Atmospheric and Oceanic Sciences and Key Laboratory of Polar Atmosphere-ocean-ice System for Weather and Climate of Ministry of Education, Fudan University, Shanghai, 200438 China; 2 Department of Atmospheric and Oceanic Sciences, School of Physics, Peking University, 100871 Beijing, China; 3 School of Geography, Nanjing Normal University, Nanjing, 210023 China; 4 State Key Laboratory of Numerical Modelling for Atmospheric Sciences and Geophysical Fluid Dynamics, Institute of Atmospheric Physics, Chinese Academy of Sciences, 100029 Beijing, China; 5 College of Earth Science, University of Chinese Academy of Sciences, 100049 Beijing, China; 6 Collaborative Innovation Center for Western Ecological Safety, College of Atmospheric Sciences, Lanzhou University, Lanzhou, 730000 China

**Keywords:** Physical oceanography, Climate and Earth system modelling

Correction to: *Nature Communications* 10.1038/s41467-024-46856-x, published online 23 March 2024

In this article the author name Jianping Huang was incorrectly written as Jiangping Huang. The original article has been corrected.

