# Supplementary material for: The role of mountains in shaping the global meridional overturning circulation
Source: Nat Commun. 2024 Mar 23;15:2602. doi: 10.1038/s41467-024-46856-x (PMC10960852; doi:10.1038/s41467-024-46856-x)
Supplement: Supplementary file 1 — Supplementary Information [file 41467_2024_46856_MOESM1_ESM.pdf]

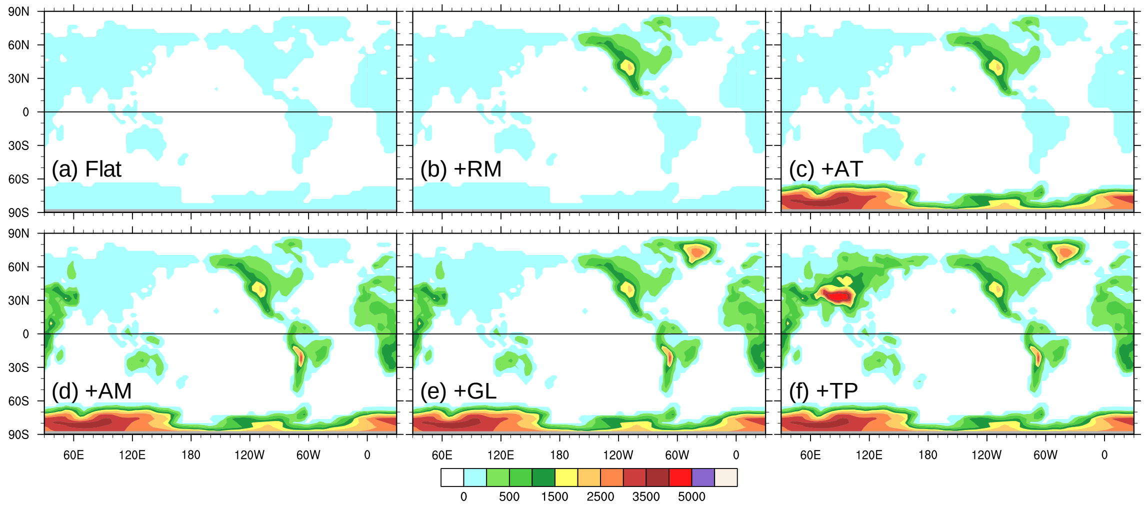


**Supplementary Figure 1 Topography configurations in coupled model experiments. a**, Modified topography with global flat topography (Flat); **b,** Modified topography after adding the Rocky Mountains (RM) to Flat; **c,** after adding the Antarctic (AT); **d,** after adding the Andes Mountains; **e,** after adding Greenland (GL); and **f,** after adding the Tibetan Plateau (TP). The "**+**" sign indicates that topography is added to the previous stage. Units: m.

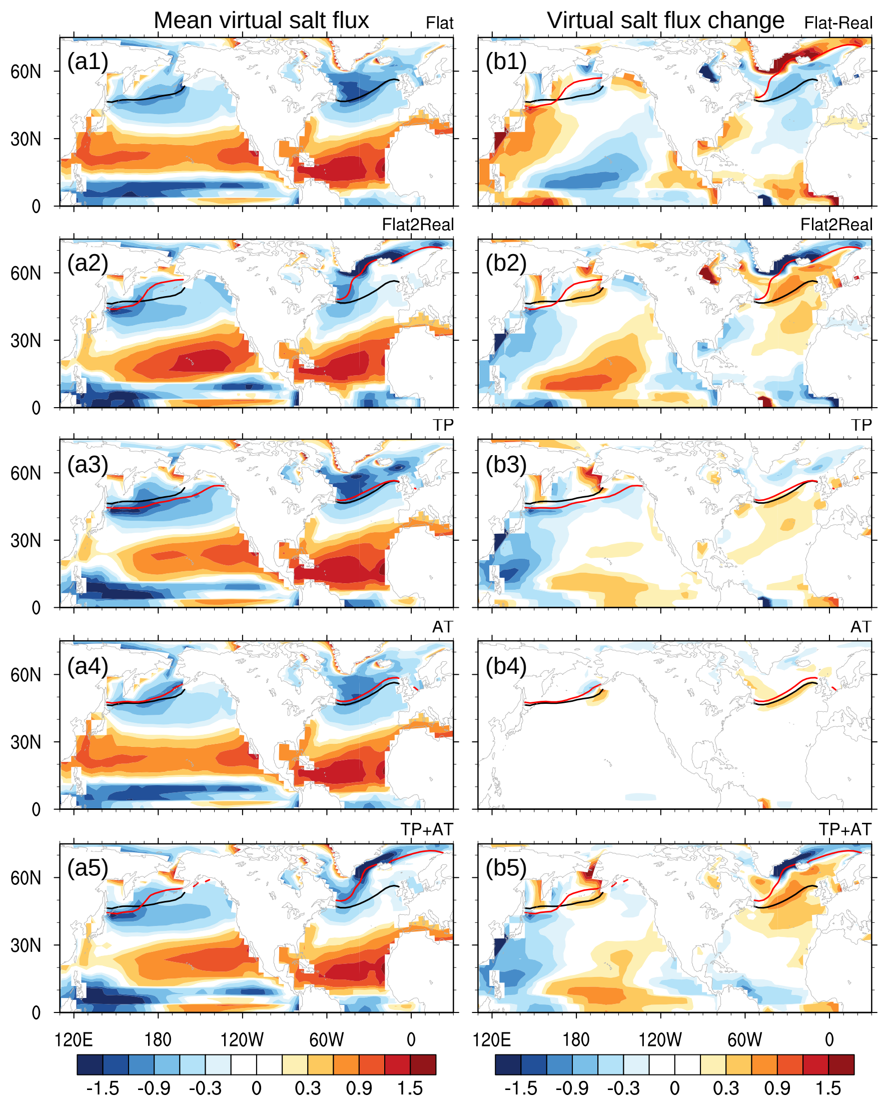


**Supplementary Figure 2 Mean VSF and its change**. **(a1-a5)**, Mean VSF (units: psu/year) in Flat, Flat2Real, TP, AT, and TP+AT, respectively. Black curve represents sea-ice margin in Flat, and red curve is for respective experiment. **b1**, Equilibrium change of VSF in Flat with respect to Real. **(b2-b5)**, Equilibrium changes of VSF in Flat2Real, TP, AT, TP+AT, and AM, respectively, with respect to Flat. Sea-ice margin is defined by 15% sea-ice fraction. Positive value denotes that the ocean becomes more saline due to VSF. Note that patterns of **b1** and **b2** are nearly identical, but have opposite signs. **b1** is included here for the convenience of analyses presented in the paper.

From Flat to TP, the pattern of VSF change (**b3**) is similar to that of Flat2Real (**b2**). However, the magnitude of this change in the North Atlantic is much weaker than that in Flat2Real. This suggests the TP uplift is sufficient to shut down the PMOC, but it can only cause marginal NADW formation, which is insufficient for the AMOC establishment.

From Flat to AT, the VSF change in the Northern Hemisphere (NH) is negligible (**b4**). This suggests the AT uplift has a minimal effect on the NH hydrological cycle, which would not alter the GMOC in Flat.

From Flat to TP+AT, the VSF change (**b5**) is nearly identical to that in Flat2Real (**b2**), in terms of both pattern and magnitude. This suggests that these two giant structures can roughly fulfill the duties of global mountains in shaping the GMOC.

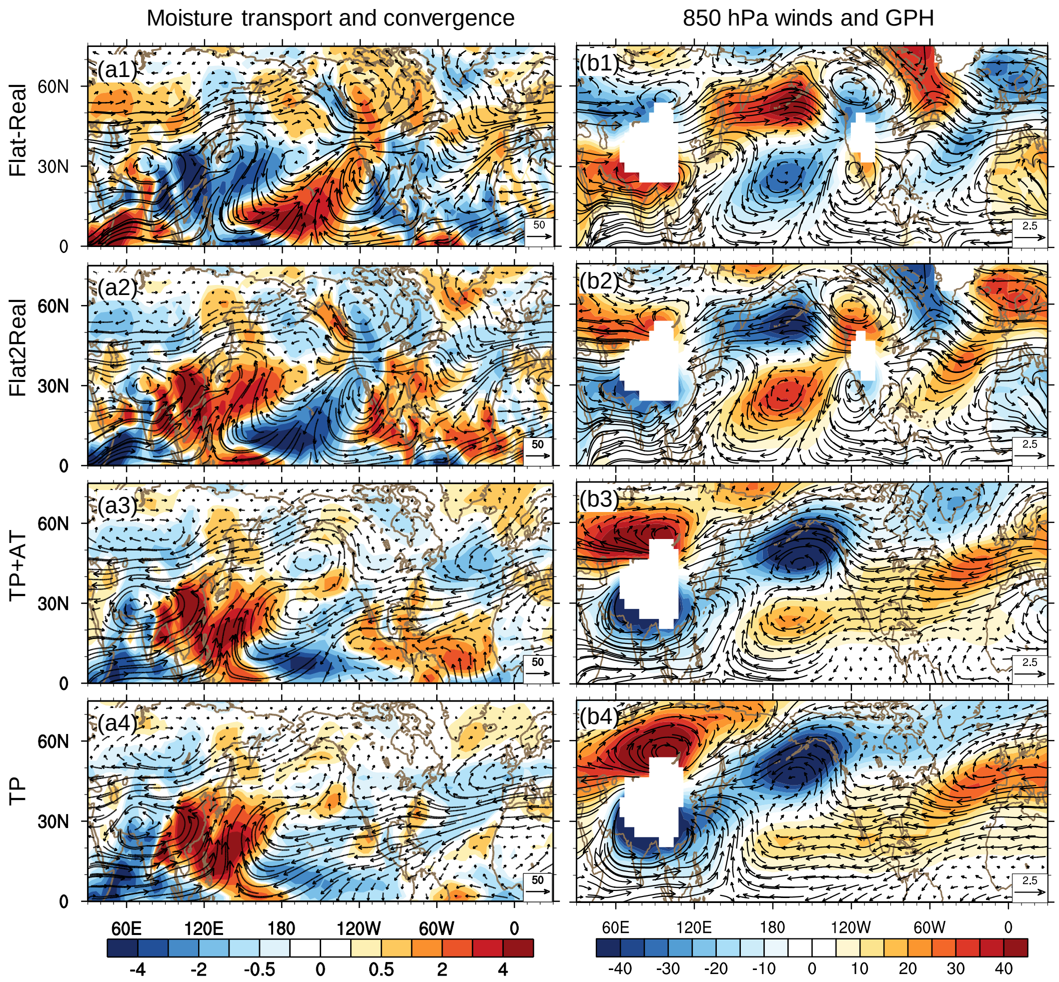


**Supplementary Figure 3 Equilibrium changes in atmospheric circulation and water vapor transport**. **(a1-a4)**, Changes in vertically integrated water vapor transport (vector; units: kg·m$^{-1}$s$^{-1}$) and its convergence (shading; units: $10^{-5}$ kg·m$^{-2}$s$^{-1}$); **(b1-b4)**, Geopotential height (shading; units: 10 m) and wind (vector; units: m/s) at 850 hPa. **a1** and **b1** are changes in Flat, with respect to Real. **(a2-a4)** and **(b2-b4)** are changes in Flat2Real, TP+AT and TP, respectively, with respect to Flat. Atmospheric water vapor convergence (divergence) is plotted as positive (i.e., $-\nabla \cdot \vec{v}q > 0$) (negative, i.e., $-\nabla \cdot \vec{v}q < 0$), representing a gain (EMP<0) (loss, EMP>0) of ocean fresh water from (to) the atmosphere. Note that patterns of **a1** and **a2** and those of **b1** and **b2** are nearly identical, but have opposite signs. **a1** and **b1** are included here for the convenience of analyses presented in the paper.

From Flat to TP and From Flat to TP+AT, the patterns of atmospheric changes (**a4**, **b4** and **a3**, **b3**) are similar to those of Flat2Real (**a2**, **b2**), particularly regarding moisture changes and wave structures over the Euro-Asian continent and the North Pacific. There are some differences between them over the eastern North Pacific, North American continent, and North Atlantic, which can be attributed to the effect of the RM. The presence of the RM does substantially affect the atmospheric circulation and moisture situation over the North American continent and North Atlantic. However, as revealed previously (Jiang and Yang, 2021), the net effect of all factors related to the RM on the VSF in the North Atlantic is small.

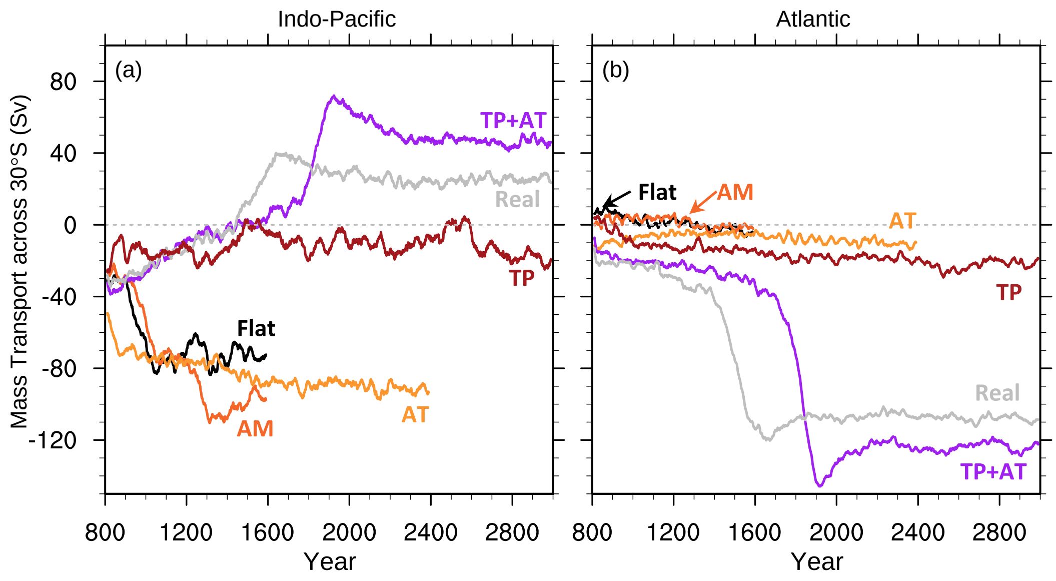


**Supplementary Figure 4 Evolution of meridional mass transport across 30°S in the intermediate-deep ocean**. **a**, **b**, Mass transports across 30°S (units: Sv) in the South Indo-Pacific and South Atlantic, respectively. The mass transport is obtained by integrating the meridional velocity over the depth of 2000-3000 m along 30°S. Positive (negative) value denotes northward (southward) transport.

In the present climate (Real), the strong thermohaline circulation in the Atlantic corresponds to a strong southward mass transport in the intermediate-deep ocean of the South Atlantic, while the strong wind-driven circulation in the Indo-Pacific corresponds to a moderate northward mass transport in the South Indo-Pacific, as depicted by the grey curve in **b** and **a**, respectively.

In Flat, the strong southward mass transport occurs in the South Indo-Pacific due to the PMOC, while the corresponding mass transport in the South Atlantic is very weak due to weak wind-driven circulation, as depicted by the black curve in **a** and **b**.

In Exp TP, the PMOC is shut down and the AMOC is not fully established, resulting in weak meridional mass transports in both the South Indo-Pacific and Atlantic, as depicted by the dark-red curve in **a** and **b**.

In TP+AT, the AMOC is fully established and the PMOC disappears completely. The situation of meridional mass transport is similar to that in Real, as depicted by the purple curve in **a** and **b**.

In Exps AT and AM, the situations are similar to those in Flat.

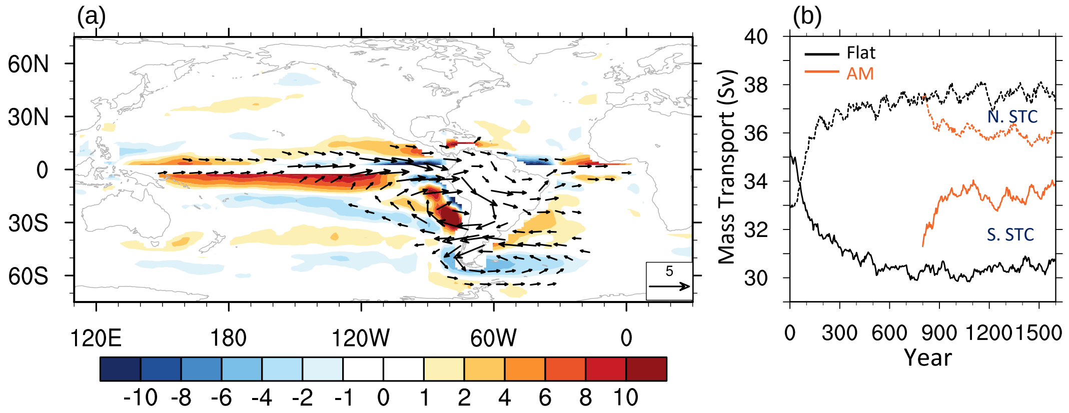


**Supplementary Figure 5 Equilibrium changes in wind and Ekman pumping, and temporal evolution of the STCs in AM with respect to Flat**. **a**, Changes in surface wind (vector; units: m/s) and Ekman Pumping (shading; units: cm/day) with positive (negative) value for upwelling (downwelling). **b**, Evolution of STC (units: Sv) in Flat (black curve) and Exp AM (red curve) in the North Pacific and South Pacific, respectively. The dashed (solid) curves represent the northern (southern) STC, whose index is defined as the absolute maximum value of the STC in the upper 500 m between 0° and 30°N (30°S and 0°).

Compared to Flat, the stronger southern STC in Exp AM (solid orange curve in **b**) is a result of stronger Ekman pumping in the tropical Pacific south of the equator (as depicted in **a**), which reinforces the southern branch of the PMOC in the South Pacific. The weaker northern STC in the North Pacific is due to the counteraction between the lower branch of the northern STC (southward) and the upper branch of the PMOC (northward).

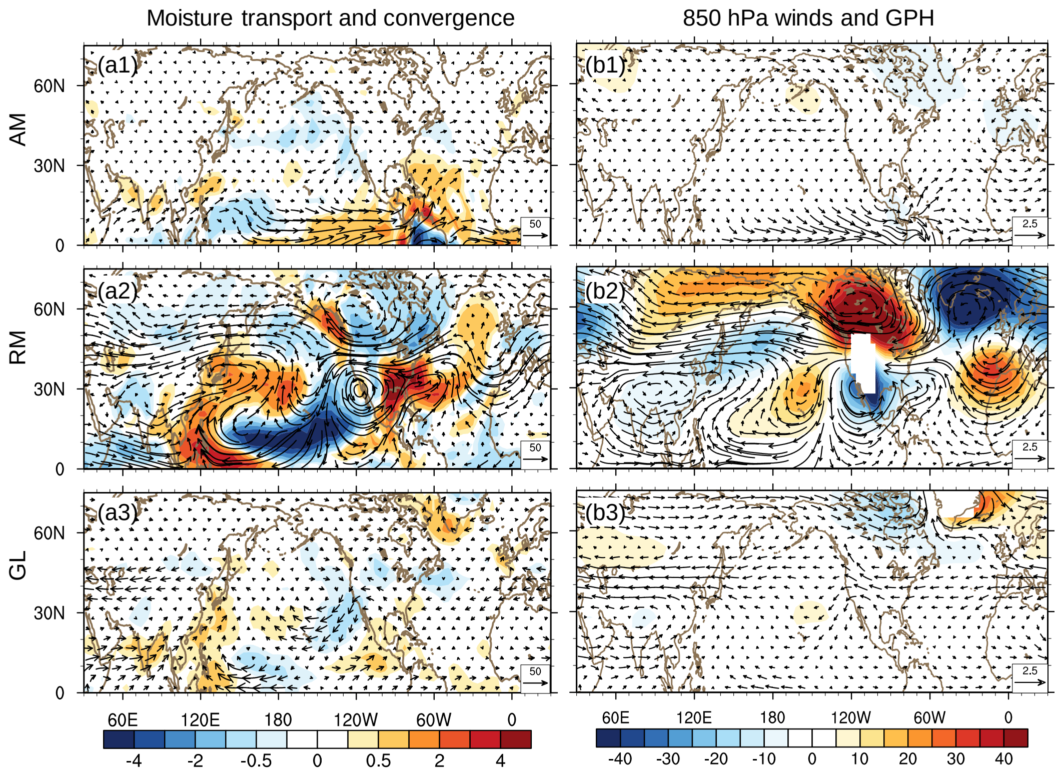


**Supplementary Figure 6 Equilibrium changes in atmospheric circulation and moisture transport**. **(a1-a3)** changes in vertically integrated moisture transport (vector; units: $kg{\cdot}m^{-1}s^{-1}$) and its convergence (shading; units: $10^{-5}$ $kg{\cdot}m^{-2}s^{-1}$), **(b1-b3)** geopotential height (shading; units: 10 m) and wind (vector; units: m/s) at 850 hPa. **(a1-a3)** and **(b1-b3)** are changes in Exps AM, RM, and GL, respectively, with respect to Flat. The atmospheric moisture convergence (divergence) is plotted as positive (i.e., $-\nabla \cdot \vec{v}q > 0$) (negative, i.e., $-\nabla \cdot \vec{v}q < 0$), representing a gain (EMP<0) (loss, EMP>0) of ocean fresh water from (to) the atmosphere.

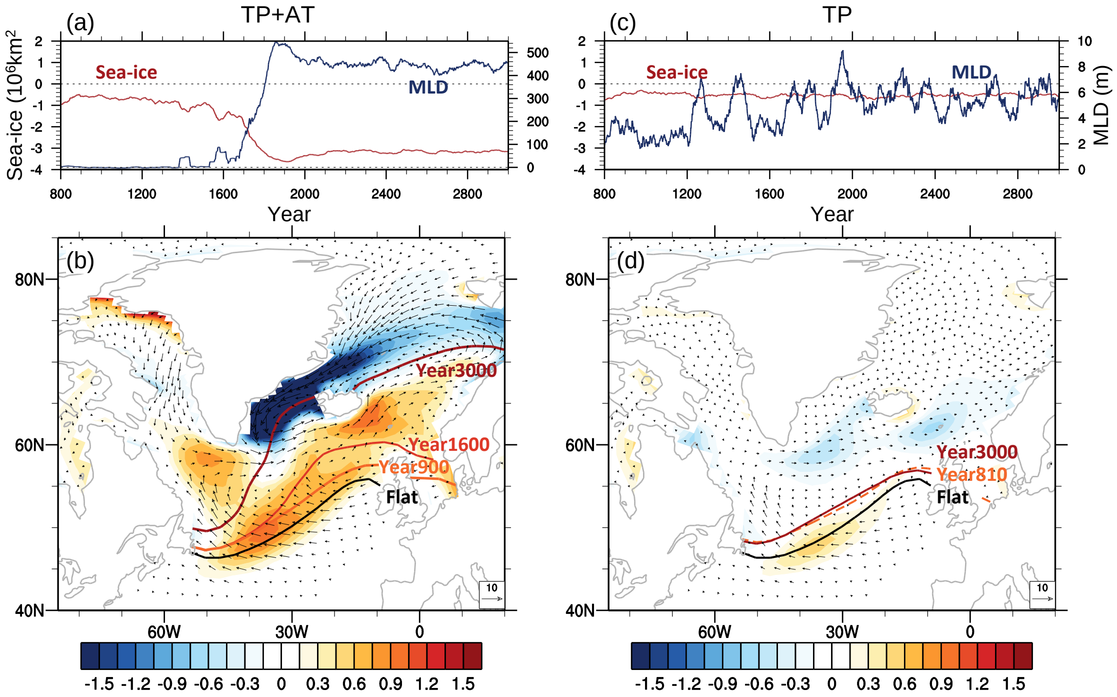


**Supplementary Figure 7 Changes in sea ice and MLD. (a, b)** are for TP+AT; and **(c, d)** for Exp TP. **a, c**, Evolutions of changes in sea-ice cover in the Arctic (units: $10^6$ km$^2$, left ordinate) and MLD in the subpolar Atlantic (units: m, right ordinate), with respect to Flat. The sea-ice cover is annual averaged. The MLD is for March and is calculated using the method of Large et al. (1997), which represents the site of the deepest vertical mixing and convection, and thus deep-water formation. **b, d**, Sea-ice margins (curve) at different stages, and equilibrium changes in VSF due to sea-ice formation or melting (shading; units: psu/year) and sea-ice velocity (vector; units: cm/s), with respect to Flat. Sea-ice margin is defined by 15% sea-ice fraction; and different colors show sea-ice margins in different stages of the uplift. Positive (negative) VSF means the ocean loses (gains) fresh water.

In TP+AT, the sea-ice retreat and MLD deepening in the subpolar Atlantic are similar to those in Flat2Real. However, in Exp TP, the sea ice retreats only slightly, and the MLD remains nearly unchanged.
